# Studying Metabolism by NMR-Based Metabolomics

**DOI:** 10.3389/fmolb.2022.882487

**Published:** 2022-04-27

**Authors:** Sofia Moco

**Affiliations:** Division of Molecular and Computational Toxicology, Department of Chemistry and Pharmaceutical Sciences, Amsterdam Institute for Molecular and Life Sciences, Vrije Universiteit Amsterdam, Amsterdam, Netherlands

**Keywords:** metabolomics, NMR, metabolism, qNMR, stable isotopes, metabolite-protein interactions

## Abstract

During the past few decades, the direct analysis of metabolic intermediates in biological samples has greatly improved the understanding of metabolic processes. The most used technologies for these advances have been mass spectrometry (MS) and nuclear magnetic resonance (NMR) spectroscopy. NMR is traditionally used to elucidate molecular structures and has now been extended to the analysis of complex mixtures, as biological samples: NMR-based metabolomics. There are however other areas of small molecule biochemistry for which NMR is equally powerful. These include the quantification of metabolites (qNMR); the use of stable isotope tracers to determine the metabolic fate of drugs or nutrients, unravelling of new metabolic pathways, and flux through pathways; and metabolite-protein interactions for understanding metabolic regulation and pharmacological effects. Computational tools and resources for automating analysis of spectra and extracting meaningful biochemical information has developed in tandem and contributes to a more detailed understanding of systems biochemistry. In this review, we highlight the contribution of NMR in small molecule biochemistry, specifically in metabolic studies by reviewing the state-of-the-art methodologies of NMR spectroscopy and future directions.

## Introduction–NMR, a Toolset of Strategies in Studying Metabolism

Nuclear Magnetic Resonance (NMR) is a spectroscopic technique that takes advantage of the energetic transition of nuclear spins in the presence of a strong magnetic field. Since the first NMR spectrum published in 1940s, the use of NMR as an analytical chemistry discipline has matured into numerous areas (Claridge, 2006). NMR has proven to be an essential tool in life sciences including in the identification and structure elucidation of organic molecules and specifically metabolites; in studying the dynamics of macromolecules such as proteins and nucleic acids; and more recently in the field of metabolomics (Cohen et al., 1995; Vignoli et al., 2019). Because NMR measurements of molecules are so sensitive to the chemical environment, it offers selective chemical information about molecules in their physiological setting.

The use of NMR in metabolic studies has a long history. ^31^P NMR was firstly used to monitor phosphorous-containing metabolites, such as nucleotide and sugar phosphates, including redox species, in cells and tissues (Hoult et al., 1974; Shulman et al., 1979; Gadian and Radda, 1981). Researchers in the late 1970s optimistically stated ‘it is now possible to obtain on metabolites *in vivo* the kinds of detailed information about structure, motion, reaction rates, and binding sites that have been obtained by NMR studies of purified biomolecules in solution’. (Shulman et al., 1979). Many of these topics are still the subject of research using NMR methodologies today.

Radioactive tracers were the gold standard in studying metabolic fate of molecules in biological systems with widespread application in fields such as medicine, nutrition, toxicology, environmental sciences, and pharmacology. Radioactive isotopes have been progressively replaced by safe stable isotope tracers with the development of labelled supplies and improved detection strategies (Matwiyoff and Ott, 1973; Jang et al., 2018). Stable isotope resolved metabolomics (SIRM) can determine activities of many metabolic reactions across a wide variety of metabolic pathways and has been used to determine absolute metabolic fluxes (Buescher et al., 2015; Lane et al., 2019). Mass spectrometry (MS) and NMR are the techniques of choice in analysing labelling experiments (Lane et al., 2019). NMR is able to provide positional labelling information, a recognisable advantage in discerning metabolite information (Fan et al., 2012).

The establishment of a new era of biological mixture analyses - metabolomics - has emerged because of the development of advanced technologies. Confidence in measuring metabolites has become widespread. These technological advancements, in addition to the pressing societal need in understanding metabolic diseases, boosted a refreshed interest in metabolic studies over the past decade. After all, metabolism pervades every aspect of biology (Deberardinis and Thompson, 2012). While mass spectrometry (MS) has been adopted in many laboratories for metabolic and metabolomics studies because of its wide coverage and high sensitivity, NMR remains used by a smaller community of scientists. NMR gathers several advantages (Emwas et al., 2019). NMR measurements are highly robust: inter-laboratory measurements are reproducible (Ward et al., 2010) and the stability of instrumental response can be months to years if samples are appropriately stored (Pinto et al., 2014). In regular NMR experiments, samples are in tubes and no chromatographic methods are used: hence, the sample is not in contact with the instrument eliminating the need for cleaning the instrument. NMR spectrometers can easily be shared among users with diverse applications, without risk of contamination or carry-over. NMR is quantitative, so both relative and absolute metabolite concentrations can be obtained. Most isomers lead to distinct spectra, making NMR an indispensable tool in structure elucidation.

**FIGURE 1:**
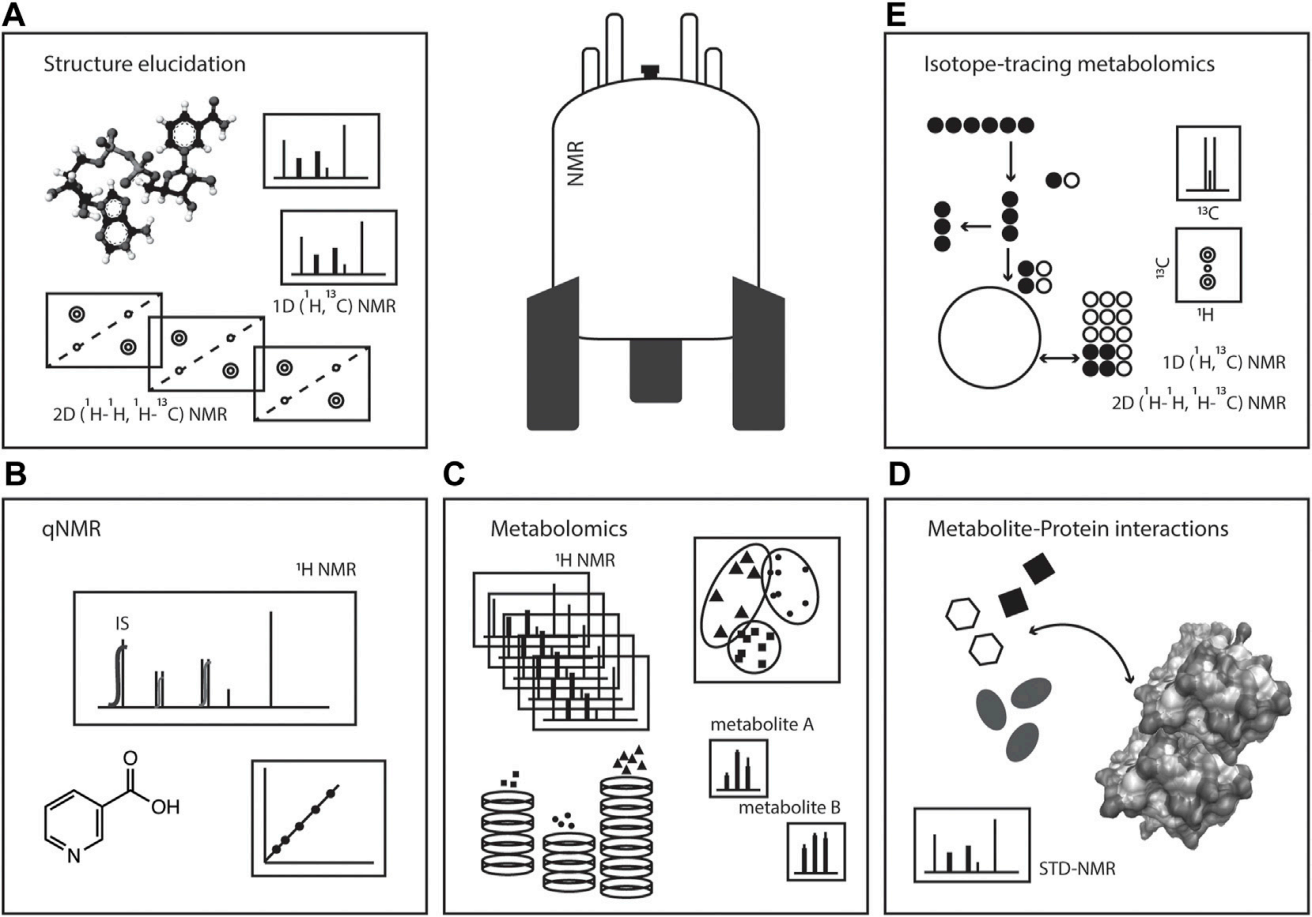
NMR spectroscopy: a toolset in metabolism studies. Pictorial representation of the various ways NMR spectroscopy can be used in metabolic studies, such as **(A)** structure elucidation, **(B)** quantitative NMR (qNMR), **(C)** metabolomics, **(D)** metabolite-protein interactions, and **(E)** isotope-tracing metabolomics or stable isotope resolved metabolomics (SIRM).

In this review, we will focus on several NMR strategies of interest in studying metabolism: i) metabolomics analyses; ii) metabolite identification and structure elucidation; iii) quantification (qNMR) of metabolites; iv) the use of stable isotopes in metabolism studies; and v) metabolite-protein interactions, Figure 1. The versatility of NMR makes this spectroscopy a powerful toolset in tackling metabolism questions in a variety of biological systems, aiding in unravelling fundamental aspects of biochemistry including metabolite identification, quantification and turnover, metabolic activities, organelle compartmentalisation, and metabolite interaction with macromolecules for enzymology or regulatory events.

## NMR Metabolomics and Metabolic Profiling

**FIGURE 2:**
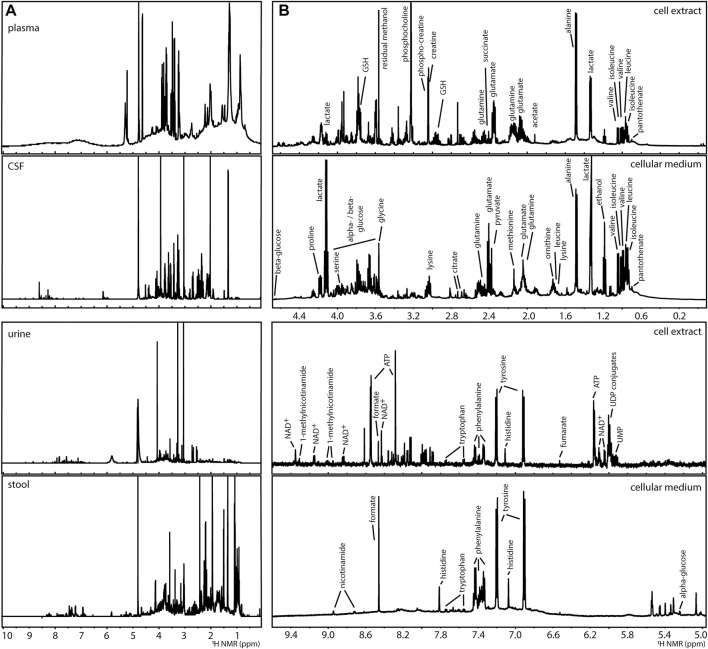
Examples of ^1^H NMR spectra of metabolomics analyses of **(A)** human biofluids (prepared in phosphate buffer saline in D_2_O pH 7.4): plasma, cerebral spinal fluid (CSF), urine, and extract of faecal water (stool) and, **(B)** a human liver cell model (HepG2) after 24 h of culture: intracellular content (cell extract prepared by methanolic extraction) and extracellular content (cellular medium), indicating some of the detected metabolites.

The analysis of complex mixtures (as in metabolomics) by NMR has been used in the characterisation of foods, natural extracts, and biological samples (Moco et al., 2007; Larive et al., 2015; Hatzakis, 2019). A variety of biological samples, such as extracts of microorganisms from the gut microbiome (Klünemann et al., 2021), mammalian cell systems (Kostidis et al., 2017), mammalian (Beckonert et al., 2007) and plant (Kim et al., 2011) tissues, and clinical tissues and biofluids such as plasma, urine, cerebral spinal fluid or faecal water (Beckonert et al., 2007; Martin et al., 2012; Da Silva et al., 2013) have been described, Figure 2A. ^1^H NMR spectra, such as NOESY-1D (1D Nuclear Overhauser Effect Spectroscopy), are commonly utilised generating catalogues of profiles of a large number of metabolites. About 60 metabolites can be identified in an untargeted ^1^H NMR spectrum using a 600 MHz NMR spectrometer in samples (such as human urine) with little effort in sample preparation (Takis et al., 2017; Vignoli et al., 2019). The ^1^H NMR analysis of blood matrices such as serum, in addition to small molecules (metabolites) also allows for the detection of lipoprotein classes (Soininen et al., 2009). For example, the analysis of a human cell system detects amino acids, organic acids, sugars, and other metabolites mainly belonging to central carbon metabolism and connected pathways, Figure 2B (Kostidis et al., 2017). While in most metabolomics applications, biological samples are placed in solution, analysis of intact tissues can be done by high resolution (HR) magic angle spinning (MAS)-NMR (Chan et al., 2009). However, all obtained ^1^H NMR spectra in metabolomics suffer from considerable signal overlap since sample preparation is minimal, each metabolite often leads to several signals in the spectrum, and many metabolites can be detected.

The use ^1^H NMR spectra for metabolomics requires consistent solvent suppression and a flat baseline. Since many biological matrices are water-based, suppressing the solvent signal allows for a better detection of lower abundant compounds and increased sensitivity. Solvent suppression also decreases radiation damping in cryoprobes (Barding et al., 2012). Although there are different pulse sequences that suppress solvent signals, such as WET, WATERGATE or PURGE, NOESY-1D with presaturation and Carr-Purcell-Meiboom-Gill (CPMG) are probably the most widely used in metabolomics (Giraudeau et al., 2015). A flat baseline is essential for subsequent statistical analysis and metabolite quantification (Barding et al., 2012; Emwas et al., 2015; Giraudeau et al., 2015).

While 2D NMR experiments such as ^1^H,^13^C-Heteronuclear Single Quantum Coherence (HSQC) (Bingol et al., 2014), ^1^H-^1^H-Total Correlation Spectroscopy (TOCSY) (Jiang et al., 2020), 2D-^1^H-*J*-resolved (JRes) or 2D-^1^H-Diffusion-ordered NMR spectroscopy (DOSY) or Concentration-ordered NMR spectroscopy (CORDY) (Huang et al., 2015) offer a more deconvoluted picture of a mixture, these experiments take considerably more time and are computationally more intensive to process. Consequently, 2D NMR experiments are infrequently used for fingerprinting purposes in large studies. Even though ^1^H NMR is the mostly widely nucleus used in metabolomics, other nuclei such as ^13^C (Clendinen et al., 2015), ^15^N (Bhinderwala et al., 2018) and ^31^P (Bhinderwala et al., 2020) have been applied in direct NMR analyses. These nuclei are usually studied through ^1^H magnetisation in 2D NMR experiments. The ubiquity of ^1^H in most metabolites and its high NMR sensitivity make ^1^H NMR the ideal nucleus in NMR-based metabolomics.

An important advantage of NMR-based metabolomic studies is the reproducibility among laboratories (Ward et al., 2010). Given the robustness of the NMR measurement, standardisation of procedures has become progressively easier, especially in clinical applications such as the analysis of human urine, blood serum and plasma. Urine samples are obtained (of course) non-invasively which has led to the development of research and clinical diagnostics. Standardisation of procedures is essential for clinical applications (Emwas et al., 2015). The speed and robustness of sample biomarker profiling with NMR spectroscopy has been extended to thousands of samples. For example, human blood plasma samples of approximately 121,000 participants from UK Biobank have been analysed, leading to an extended clinical chemistry panel consisting of 249 biomarkers and ratios, based on metabolite signals of lipoproteins, lipids, amino acids and a few glycolysis intermediates (Ritchie et al., 2021).

Since metabolomics relies on the comparative analysis of a system challenged by a perturbation relative to its control, it can be applied to a many biochemical questions related to metabolism: such as drug-induced metabolic perturbations (Vinaixa et al., 2011), aetiology of metabolic diseases, like cancer (Vignoli et al., 2021), or cellular development and differentiation (Moussaieff et al., 2015).

## Metabolite Structure Verification and Elucidation

**FIGURE 3:**
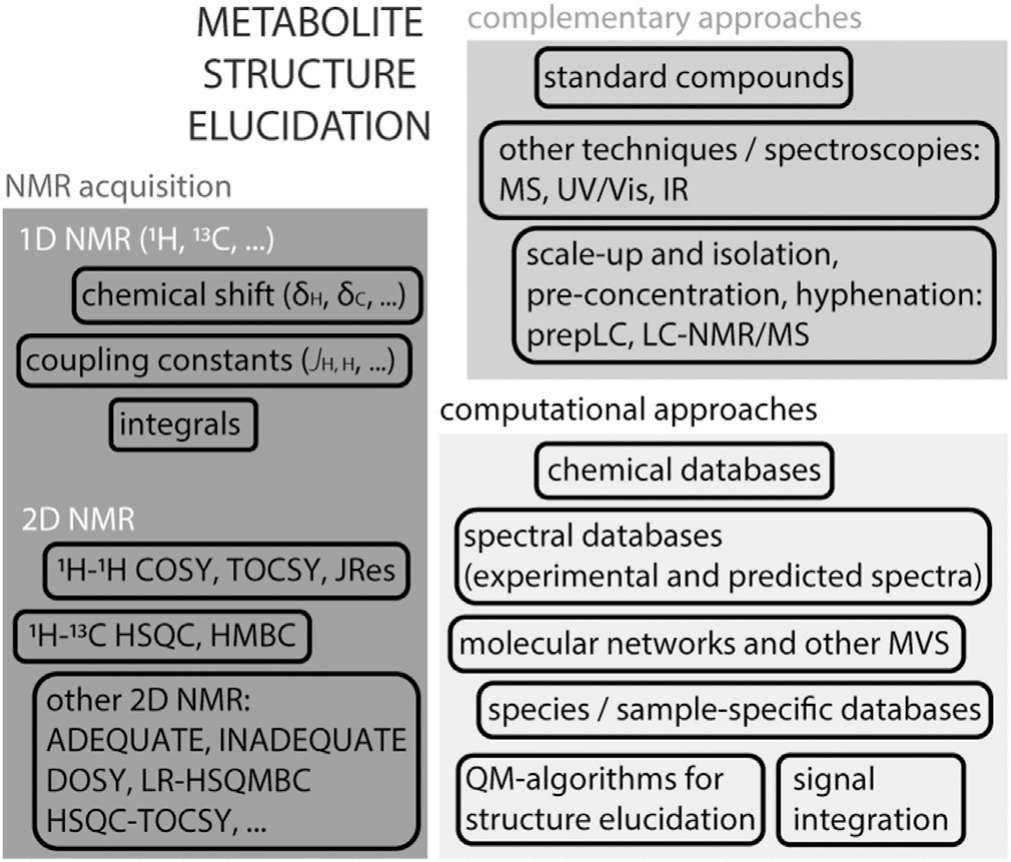
Structure elucidation strategies using NMR. Acquisition of a ^1^H NMR spectrum on an isolated compound provides essential information about the molecule’s structure such as the chemical shift (electronegativity of neighbouring protons and possible functional groups), coupling constants (multiplicity of signals reflects the influence of neighbouring protons), signal integral (assessment of equivalent protons). Atom connectivity can be assessed by homonuclear and heteronuclear 2D NMR spectra, usually ^1^H–^1^H or ^1^H-^13^C. In certain cases, other 2D or 3D NMR experiments are useful to obtain more detailed information. Identification and spectral deconvolution in NMR benefits from available computational approaches, including databases, multivariate statistical approaches (MVS) and quantum-mechanic-based algorithms. And the availability of complementary information, such as the use of authentic standards or mass spectrometry is generally helpful. In the case of complex mixtures, as in metabolomics, scale-up and metabolite isolation are often unavoidable, in particular in the case of unknown metabolites.

NMR is perhaps mostly known for its ability to elucidate chemical structures of small molecules, Figure 3. A ^1^H NMR spectrum of a given molecule provides information about functional groups (position of chemical shifts), spatial or connecting protons (multiplicity of signals and coupling patterns), and number of equivalent protons (signal integrals). The interpretation of these signals can in many cases lead to an unambiguous identification of the molecule. NMR is efficient in distinguishing many isomers, by their unique spectra. The exception are enantiomers, that require chiral agents to be derivatised into diastereomers for analyses by regular NMR spectroscopy. While ^1^H NMR provides crucial and sometimes sufficient information to resolve a structure, the complexity of certain molecules requires additional strategies. The interpretation of certain 1D ^1^H NMR spectra, in particular in the presence of complex multiplicities and second order effects, can profit from quantum-chemistry algorithms (Elyashberg et al., 2016; Pauli et al., 2021). For example, the web-based Cosmic Truth (CT) software uses experimental spectra to calculate coupling constants in complex multiplets, and thereby provide higher certainty on metabolite identification in ^1^H NMR spectra (Achanta et al., 2021; Pauli et al., 2021).

The next level of obtaining structural information came with the establishment of 2D homonuclear and heteronuclear NMR pulse sequences, which obtains enhanced atom connectivity and spatial information within spin systems. While identification of most purified metabolites can be done using a combination of standard 1D NMR ^1^H and ^13^C NMR and 2D NMR (as ^1^H, ^1^H-COSY (COrrelation SpectroscopY); ^1^H, ^1^H-TOCSY; ^1^H, ^13^C-HSQC; ^1^H, ^13^C-HMBC (Heteronuclear Multiple Bond Correlation)) experiments, identifying certain molecules requires additional information because of their complexity (Elyashberg, 2015). Methods such as ADEQUATE (Adequate Sensitivity Double-Quantum Spectroscopy), INADEQUATE (Incredible Natural Abundance DoublE QUAntum Transfer Experiment), HSQC-TOCSY and LR-HSQMBC (long-range heteronuclear single quantum multiple bond correlation) aid in putting in evidence certain properties towards resolving more complex spin systems in, for example, natural compounds (Elyashberg, 2015). Pure shift NMR spectroscopy that includes methods such as PSYCHE (Pure Shift Yielded by Chirp Excitation Suppressing) collapse multiplet signals into singlets in ^1^H NMR spectra to improve spectral resolution (Foroozandeh et al., 2014). For complete structure elucidation, nested super-pulse sequences that encompass a series of existing 2D NMR methods (e.g., HMQC-HSQC-COSY-NOESY) can be used. One example is NOAH: NMR by Ordered Acquisition using ^1^H-detection (Kupče and Claridge, 2017). A sample containing 50 mM cyclosporine in benzene-d_6_ was acquired with the NOAH-5 super-sequence (that combines ^1^H-^15^N HMQC, multiplicity edited ^1^H-^13^C HSQC, ^1^H-^13^C HMBC, COSY and NOESY pulse sequences), producing five 2D spectra in one experiment in 44 min (Kupče and Claridge, 2017).

Metabolite identification by NMR benefits from additional chemical information which can be done by integration of complementary pieces of information. Mass spectrometry (MS) can assist in providing the molecular mass of a molecule, and thereby a putative molecular formula, as well as some structural information by MS/MS fragmentation. The combination of chemical information provided by NMR and MS in combination is highly efficient in metabolite identification (Moco et al., 2007). The access to online resources with experimental and/or predicted NMR spectral databases, such as HMDB (Wishart et al., 2021), BMRB (Ulrich et al., 2007) and NMRShiftDB (Steinbeck et al., 2003) are important tools to deduce possible molecules.

Metabolite identification in metabolomics can be challenging given the presence of many overlapping signals. Libraries of metabolites found in common matrices such as urine, plasma, and serum are useful resources (Wishart et al., 2021). Structure verification in metabolomic studies is done by comparing profiles to standards in spectral databases, as well as acquisition of 2D NMR and MS directly on mixtures. The integration of NMR and MS analyses can provide confirmatory and complementary information on the underlying metabolites, avoiding metabolite isolation (Moco et al., 2008). Multivariate statistical tools as Statistical Total Correlation Spectroscopy (STOCSY) (Cloarec et al., 2005), Subset Optimization by Reference Matching (STORM) (Posma et al., 2012) or Resolution EnhanceD SubseT Optimization by Reference Matching (RED-STORM) (Posma et al., 2017), have been used in biofluid spectra, highlighting spectral regions of differential metabolites. The multiple resonances of a metabolite can be correlated across metabolite datasets.

Concentrating the sample or compound isolation prior to NMR analysis in inevitable when dealing with unknown molecules or unknown matrices. Hyphenated techniques, such as liquid chromatography (LC)-NMR-MS or LC-solid phase extraction (SPE)-diode array detection (DAD)-MS/NMR (Moco and Vervoort, 2012; Garcia-Perez et al., 2020) have been developed. For example, the identification of the uremic toxins *N* ^1^-methyl-2-pyridone-5-carboxamide and *N* ^1^-methyl-4-pyridone-5-carboxamide in C57BL/6 mice’s urine was possible through a combination of sequential 1D NMR, STOCSY, 2D NMR, SPE, 2D NMR and spiking of standards (Garcia-Perez et al., 2020). Complex matrices such as a plant extract or a biological sample can be separated by LC, detected by MS with the NMR spectra acquired in a subsequent integrated step (Wolfender et al., 2019). Experimental data in combination with computational tools, including chemometrics, are used in concerted ways to fully describe complex mixtures, often with few if any sample separation steps (Wolfender et al., 2019).

## Metabolite Quantification

NMR is inherently quantitative. However, for many applications, qualitative analyses suffice, and the quantitative aspect is overlooked. Quantitative NMR (qNMR) is progressively gaining attention, with applications to drugs, vaccines, natural products, and mixtures such as biological samples and plant extracts (Holzgrabe, 2010; Simmler et al., 2014; Giraudeau, 2017; Li and Hu, 2017; Giancaspro et al., 2021). The basic principle of qNMR relies on the intensity of the NMR signal of an analyte being proportional to the number of nuclei. One of the important determinants in quantitative analysis is the optimisation of longitudinal relaxation time, T1, of protons in ^1^H qNMR. To obtain truly quantitative spectra, long delays are often required to allow full proton relaxation, as the delay is set to be at least 5 times T1 to have >99.3% of protons to return to original position (Holzgrabe, 2010). For example, the T1 of maleic acid in D_2_O phosphate buffer saline pH 7.4, a commonly used internal standard in qNMR, is ∼6.5 s. If this is the longest T1 of the resonances found in the sample to quantify, the inter-scan relaxation delay should be set to >30 s. The challenging part of implementing a qNMR routine is maintaining the exactness of procedures, from consistently using the same acquisition and processing parameters to taking into account the physico-chemical properties of the sample (pH, ionic strength, solubility, chemical interactions and interferences, storage) and calibration of scales and pipets (Bharti and Roy, 2012). The quantification of pure compounds or simple mixtures is done by purity analysis and often reference materials are used. This is quite commonplace in pharmaceutical formulations. When implemented, qNMR can lead to superb results in accuracy (<1% error) and robustness (Holzgrabe, 2010; Mahajan and Singh, 2013).

qNMR in 1D-^1^H NMR of complex mixtures acquired for metabolomic studies is usually less accurate, yielding a trueness of 10–20% (Giraudeau, 2017) since many of the analytical and instrumental parameters are cannot be optimised. qNMR on 2D homonuclear and heteronuclear NMR spectra have also been reported (Giraudeau, 2017; Li and Hu, 2017), which has the advantage of additional metabolite deconvolution compared to 1D NMR. The increased use of fast 1D ^1^H NMR metabolomics analyses has generated interest in developing quantification strategies in these spectra. Internal reference signals as ERETIC and PULCON avoid the use of external references that crowd spectra (Wishart, 2008; Holzgrabe, 2010).

qNMR in metabolomics is often performed by chemometric analyses (Wishart, 2008; Madrid-Gambin et al., 2020). Metabolomic analytical procedures require high consistency to obtain (semi-) quantitative values. Standardized pre-laboratory procedures (sample collection, storage), sample preparation, spectral acquisition, pre-processing of spectra (referencing, phasing, baseline correction, etc) and statistical analyses or machine learning are all necessary to obtain quantitative results (Wishart, 2008). Signal identification and integration across a series of spectra, including either binning or dynamic integration with alignment and normalisation before quantification are common steps. Given that certain metabolite signals are likely to overlap, it is important to define the least overlapped signals as the metabolite quantifier. While other techniques such as LC-MS, require the use of labelled internal standards and laborious method development and validation, qNMR is easier to implement. Metabolites quantification within the μM to mM range is feasible in NMR metabolomics spectra. For example, the extracellular metabolites in media of mammalian cells was quantified by NMR with a ∼15% error (Kostidis et al., 2017). Lineshape fitting models have been used in deconvoluting metabolites in ^1^H NMR spectra of ultrafiltrated human serum samples, integrating 42 metabolites and explaining >92% of the spectrum (Mihaleva et al., 2014). Computational strategies for qNMR are likely to be further developed for metabolite quantification especially for clinical research studies and acceptance for use in clinical diagnostics.

## Stable Isotope Resolved Metabolomics

Metabolite analysis of cells, organelles as mitochondria, and organs was initiated by Shulman and co-workers in 1970s. By using ^31^P and ^13^C NMR they were able to study aspects of cellular metabolism as oxidative phosphorylation and kinetics of glycolysis in *E. coli* and rat liver cells (Radda and Seeley, 1979; Shulman et al., 1979). Metabolic networks are often highly homeostatic and branched making it difficult to understand metabolic regulation by assessing metabolite concentrations in steady state conditions. A more informative approach is to assess metabolite turnover, defined as the quantity of the metabolite moving through its pool per unit time (McCabe and Previs, 2004; Fan and Lane, 2008). Metabolic turnover is studied with (usually isotope) tracers. Labelled compounds allow the determination of rates of a metabolic flux. Stable isotopes have largely replaced radioactive tracers and use MS or NMR as technologies.

NMR is particularly useful in metabolic studies because it can provide quantitative information of many metabolites at the same time as well as to distinguishing positional labelling. Stable isotope tracer analysis (or stable isotope resolved metabolomics, SIRM) by NMR commonly uses ^13^C, but also ^15^N (Lapidot and Gopher, 1997) or ^2^H (Mahar et al., 2020) labelled tracers. Different tracers may be used according to the pathway of interest. For example [^13^C_1,2_]-glucose can be used to distinguish between oxidative and non-oxidative branches of the pentose phosphate pathways because of the distribution of ^13^C in different downstream metabolites; [U-^13^C]-Glutamine is used to study glutaminolysis, as well as TCA cycle, amino acid metabolism and pyrimidine biosynthesis; and [U-^13^C]-palmitic acid is often used to study ß-oxidation (Fan and Lane, 2008; Jang et al., 2018; Saborano et al., 2019), Figure 4. Since NMR allows to measure isotopomers and metabolites in biological samples, compartmentalisation and exchange dynamics of metabolic pools can be studied. Spatial and temporal events are fundamental to understand metabolism of a given system (Fan and Lane, 2008).

**FIGURE 4:**
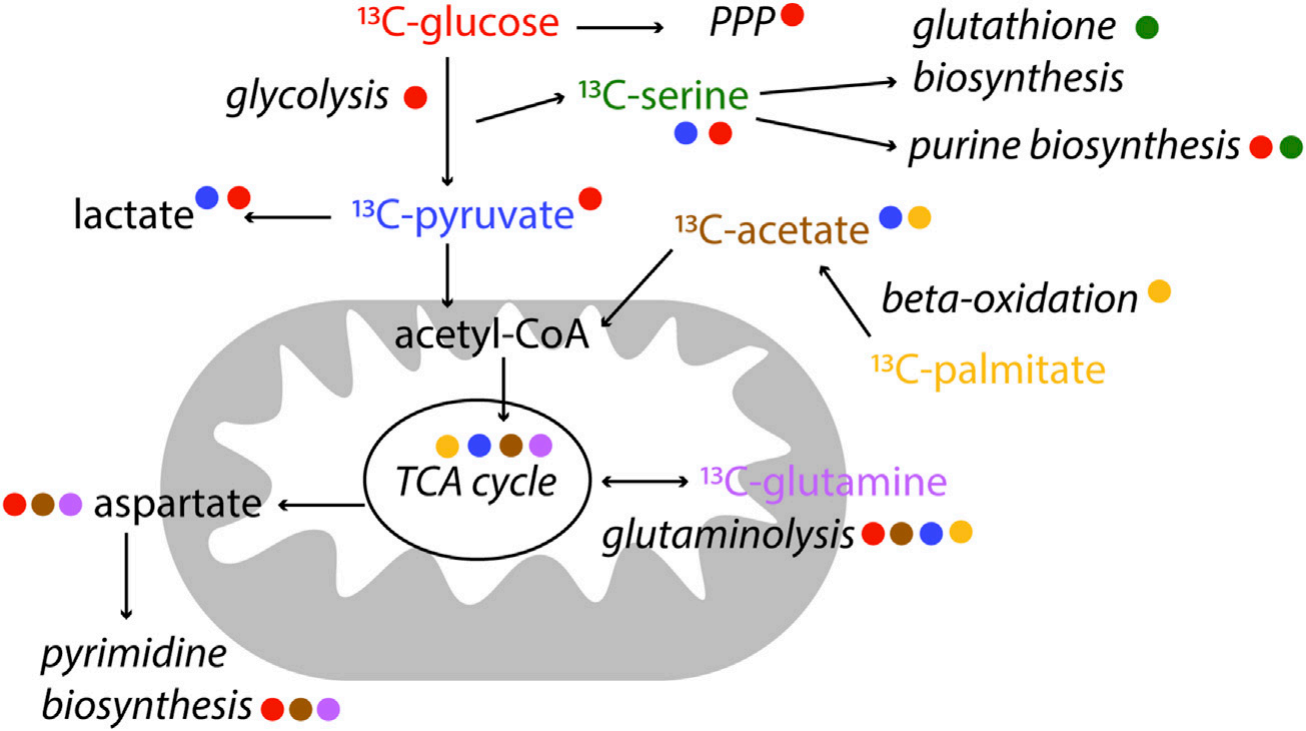
Examples of label incorporation schemes in central metabolism by NMR. Certain tracers are better suited to study specific pathways. In this scheme, the colour of the tracer is indicated next to the pathway name it is used for. ^13^C-tracers are represented, however alternative tracers with other labelled (^2^H, ^15^N) nuclei might be used, as well as other available labelled precursors.

The majority of isotopomer analysis in NMR makes use of ^13^C tracers for direct measurements of labelled carbons. Specifically, direct 1D ^1^H NMR experiments allow detecting ^13^C satellite signals, enable isotopomer distribution analysis, and are generally used for quantification of label incorporation (Fan and Lane, 2008; Vinaixa et al., 2017). Since labelled and non-labelled signals are detected in crude cell extracts, the ^1^H NMR spectrum is often crowded because of the number of metabolites present. Therefore 2D homonuclear and heteronuclear NMR experiments are used to detect characteristic labelling patterns (COSY, TOCSY, HSQC, HMBC and HCCH-TOCSY and HSQC-TOCSY) (Fan et al., 2005; Fan and Lane, 2008; Lane and Fan, 2017). ^1^H–^13^H HSQC are regularly used for ^13^C metabolic flux analysis, even if there is a large range of coupling constants (typically 120–210 Hz) in the metabolites detected (Reed et al., 2019). To make up for lengthy acquisition times of 2D NMR experiments, ultrafast 2D NMR has been applied to specific isotopic enrichments in complex biological mixtures, considerably reducing acquisition times (Giraudeau et al., 2011). ^13^C-filtered 1D spectra (and 2D spectra to reduce signal overlap) appear to be accurate in calculating label incorporation in sparsely labelled metabolic samples (Reed et al., 2019). Combining MS and NMR-based SIRM can be a strategy to obtain isotopomer distributions in a model-free way and a wider coverage of the involved intermediates (Chong et al., 2017).

Stable isotope administration can be combined with magnetic resonance spectroscopy (MRS), allowing for *in vivo* metabolic phenotyping in pre-clinical and clinical settings (Leftin et al., 2013). The use of dynamic nuclear polarization (DNP) has offered a major advantage in achieving metabolic studies *in vivo*, as it leads to an outstanding increase of sensitivity, >10,000 (Ardenkjær-Larsen et al., 2003), by using hyperpolarised substrates. For example, the use of hyperpolarized [^13^C_1_]pyruvate allowed to study skeletal muscle stimulation *in vivo* (Leftin et al., 2013).

Since central metabolism (including glycolysis, gluconeogenesis, TCA cycle and pentose phosphate pathway) is the heart of metabolism and bioenergetics, most SIRM is being performed on these pathways. For example, patients with early-stage non–small-cell lung cancer were infused with U-^13^C-glucose before tissue resection. Through SIRM analysis, it was determined that the cancerous tissues in these patients had enhanced pyruvate carboxylase (PC) activity over glutaminase 1 (GLS1), compared to non-cancerous tissues. Both PC and GLS1 are important enzymes in anaplerotic reactions, replenishing TCA cycle intermediates, needed in highly proliferating cells, as cancer cells (Sellers et al., 2015). An example of using NMR to trace other metabolic pathways besides central metabolism, is the comparison of two cancer cells line models (bladder, UMUC3, and prostate, PC3) to assess lipid metabolism turnover (Lin et al., 2021). The use of isotope tracers has also been discussed for studying nucleotide metabolism (Lane and Fan, 2015).

To perform SIRM, detection of metabolic intermediates is required, and of course, knowledge of the metabolic map (Kanehisa et al., 2014; Wishart et al., 2021). Reconstructions of metabolic models are valuable, even if certain aspects remain challenging such as compartmentalization and tissue specificity (Magnúsdóttir et al., 2016). Informatic tools become increasingly important for metabolic flux analysis calculations (Arita, 2003; Rahim et al., 2022).

Isotopes are also used to unravel metabolic fate of certain drugs or unknown molecules. In this case the aim is to study ADMET (absorption, distribution, metabolism, excretion, and toxicity) of a therapeutic agent. Many pharmaceutical drugs contain ^19^F, so ^19^F NMR can be used to monitor parent compounds and resulting metabolic products (Rietjens and Vervoort, 1989; Lindon et al., 2004; Keun et al., 2008; Reid and Murphy, 2008). ^13^C- or ^15^N-labelled drugs can also be analysed for metabolic fate using 1D- and 2D-NMR in cells and animals (Fan and Lane, 2008; Mutlib, 2008; Fan et al., 2012).

## Metabolite-Protein Interactions

Interactions between metabolites and proteins are a pre-requisite in enzymatic and allosteric events, defining metabolism and its regulation. While many methodologies to study interactions between macromolecules (e.g., protein-protein interactions) have been developed, methods to systematically assess protein-metabolite interactions are still scarce and often limited to hydrophobic metabolites (Li et al., 2010; Nikolaev et al., 2016; Piazza et al., 2018). NMR has a long history of studying protein dynamics *in vitro*, including changes in protein conformation upon ligand binding (Cohen et al., 1995) specifically by monitoring amino acid residues in the protein backbone. However, a set of ligand-observed NMR experiments can be used to specifically monitor the binding event via the ligand (in opposition to monitoring the protein). Saturation transfer difference (STD) (Mayer and Meyer, 1999; Viegas et al., 2011), Figure 5, water−ligand observation with gradient spectroscopy (WaterLOGSY) (Dalvit et al., 2001), time constant of spin-lattice relaxation in rotating frame (T1rho) and CPMG (Gossert and Jahnke, 2016) are some examples of NMR methods to monitor ligand binding to purified (non-isotopically labelled) proteins (Gossert and Jahnke, 2016). Ligand-observed NMR has been primarily used in high throughput fragment screening conducted for drug discovery (Pellecchia et al., 2008; Gossert and Jahnke, 2016).

**FIGURE 5:**
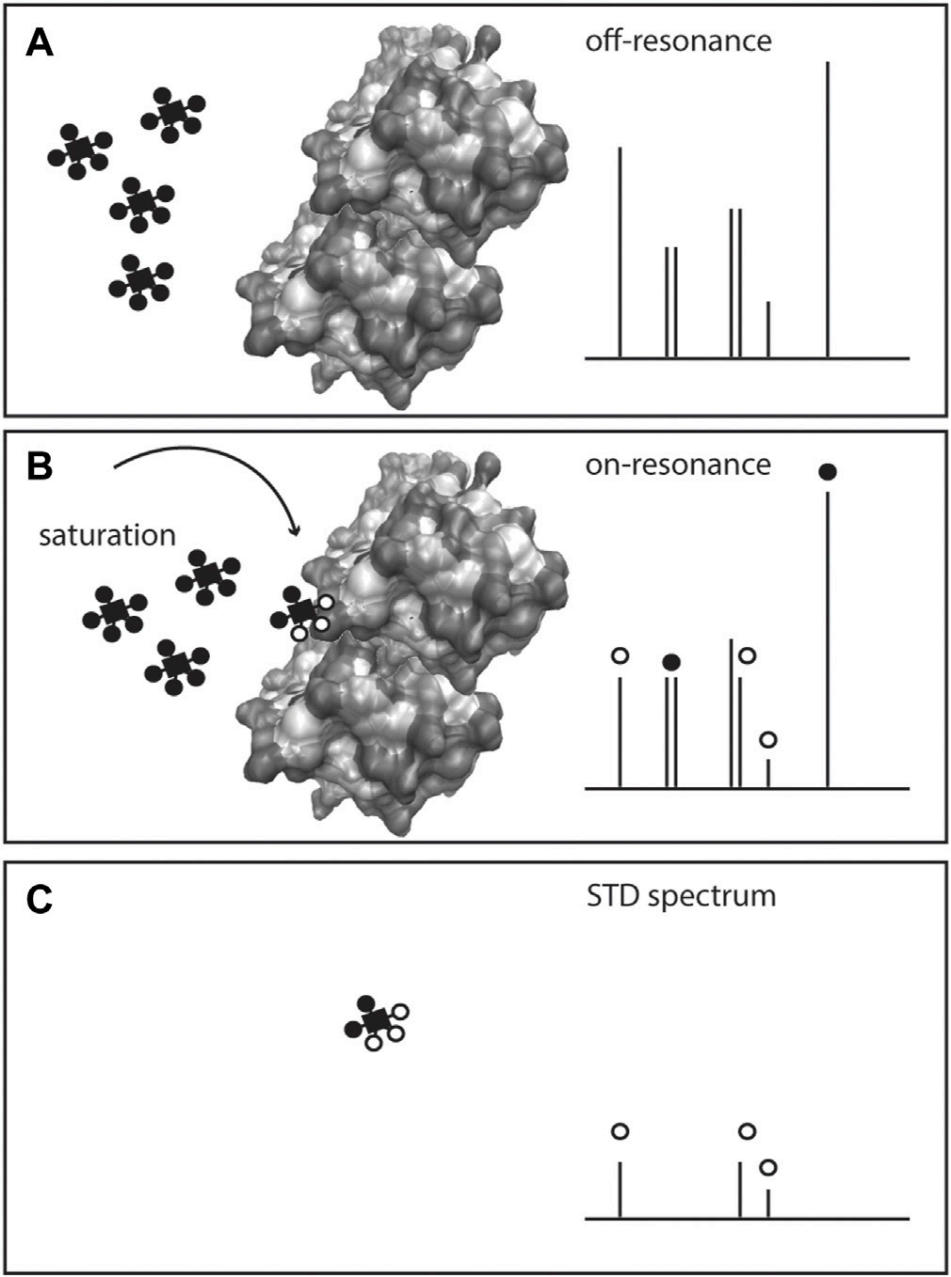
Scheme of Saturation Transfer Difference (STD)-NMR for studying protein-metabolite interactions. The protein is exposed to an excess of ligand(s) or metabolite(s) and a ^1^H NMR spectrum is recorded (off-resonance spectrum), **(A)** Given the low amount of protein in solution, only the metabolite(s) signals are visible. On a second acquisition, selected saturation is applied to the protein that is transferred to the bound metabolite through the nuclear Overhauser effect, inducing the bound ligand resonances to broaden or disappear (on-resonance spectrum), **(B)** The difference spectrum (off-resonance on-resonance), STD spectrum, **(C)** exhibits the resonances of the metabolite bound to the protein, and confirms the presence of the protein-metabolite interaction.

Ligand-observed NMR methods depend on certain conditions. The ligand is added in excess (10–20 fold to the protein amount) to a large protein (>30 kDa), and the interactions are typically weak, with dissociation constants (*K*_D_) 1 μM-10 mM. The ligand is in a fast exchange with the protein, and upon binding, the signal experiences a strong relaxation as evidenced by proton signal broadening or disappearance from the spectrum, Figure 5. By analysis of bound and unbound states, binding can be obtained via NOEs (STD and water-mediated NOEs, WaterLOSGY) on ligand signals (Meyer and Peters, 2003; Gossert and Jahnke, 2016). Calculation of *K*_D_ and even epitope mapping of the ligand interaction can be obtained. Competition between ligands in ligand mixtures can also be assessed (Viegas et al., 2011; Monaco et al., 2017).

Ligand-observed NMR has been applied to the systematic identification of endogenous metabolites-protein interactions, an example of which are the central carbon metabolism proteins of *E. coli* (Nikolaev et al., 2016; Diether et al., 2019). Solutions of up to 55 metabolites were exposed to 29 purified metabolic enzymes. This approach identified 76 novel interactions between endogenous metabolites and central metabolism enzymes (Diether et al., 2019).

While this type of approach is quite fast to set-up from the NMR acquisition side, it remains dependent on the availability of purified proteins (or at least enriched protein cell suspensions) with a defined number of metabolites. There are however efforts underway to test small molecule-macromolecule interactions in cellular environments (Siegal and Selenko, 2019). For example, whole-cell STD measurements have been used to determine the binding mode of ligands to an intracellular protein in live bacteria (Bouvier et al., 2019) and cancer cells (Primikyri et al., 2018).

## Computational Tools and Resources

Data analysis is inherent to data acquisition. Many NMR applications have been traditionally processed manually, as for example in the elucidation or confirmation of small molecule structures. However, all NMR applications benefit from computational tools and resources, for faster and more accurate extraction of biochemical information. Some of these tools and resources were mentioned in previous sections of this review. Overviews of the many publicly available resources and open source metabolomics tools have been comprehensively listed, so readers should consult them (Eghbalnia et al., 2017; Stanstrup et al., 2019; Shea and Misra, 2020).

Parsing of data into signal lists or matrices in NMR-based metabolomics is a requirement for performing multivariate statistical or machine learning analyses. This pre-processing step can be done by binning spectral data or by peak picking and integration using commercial software or public algorithms, such as, for example, AlpsNMR (Madrid-Gambin et al., 2020). ^1^H NMR spectra are sensitive to solvent, pH, ionic strength, and temperature, and thus slight shifts in proton resonances are likely to occur. This can be corrected with alignment algorithms before signal integration. There are many algorithms able to handle NMR data for various purposes, including automated putative metabolite identification according to spectral databases as HMDB (Wishart et al., 2021). Multivariate analyses or machine learning methods for NMR metabolomics spectra can be done prior to or after metabolite assignment. Typically, these analyses will assist in identifying differential significant metabolites (or metabolite features) in datasets. Details on various possibilities of handling NMR-based metabolomics data can be consulted elsewhere (Blaise et al., 2021; Debik et al., 2022). Beyond statistical treatment, web-based tools like MetaboAnalyst (Chong et al., 2018) allow to visualise metabolomics data in an user-friendly way, and are able to perform additional tasks, as for example pathway enrichment analysis (Wieder et al., 2021).

Overviews of metabolic pathways can be consulted in databases like KEGG (Kanehisa et al., 2017), HMDB (Wishart et al., 2021), WikiPathways (Kutmon et al., 2016) and Recon3D (Brunk et al., 2018) and are valuable to map metabolites from NMR spectra to specific pathways. Labelling patterns obtained from SIRM are useful for metabolic network reconstruction, however it remains challenging to fully exploit it (Lane et al., 2019).

Reports on integration of metabolome data with other omics has been attempted since genes, proteins, and metabolites collectively contribute to metabolism and its regulation. Even though multiple strategies are necessary (Jendoubi, 2021), no single or universal method is applicable for all experiments given the limitation of detections, time scales of the different omics, and specific research questions of each study.

## Summary and Future Directions

NMR spectroscopy can be used to study various aspects of metabolism. This review discussed metabolomics analyses qNMR, structure elucidation, SIRM, metabolite-protein interactions and computational approaches studied by NMR.

One of the disadvantages of NMR is its relative lower signal-to-noise, compared to other analytical techniques. The development of microprobes and cryoprobes (Anklin, 2016), as well as the use of hyperpolarised substrates by DNP (Lerche et al., 2015; Plainchont et al., 2018) are some of the strategies being used to enhance sensitivity in NMR measurements.

The development of novel methods capable of deconvoluting complex signals, such as pure shift experiments (Zangger, 2015) and fast experiments based on non-uniform sampling (Mobli and Hoch, 2014), are developments that will assist in obtaining more and faster structural information.

Efforts to standardize procedures for NMR-based metabolomics, and in particular for clinical applications, are on-going (Ritchie et al., 2021). Development of guidelines are likely to allow the use of NMR measurements as an enhanced clinical chemistry panel with applications in screening large biobanks. In this case, quantification of metabolites directly from biofluids will be essential. Hence development of computational tools for spectral deconvolution, integration and quantification will be needed.

With the worldwide increase of metabolic diseases, studying metabolic turnover of pathways through SIRM is likely to be more frequently used in research and clinical settings. Thus, diversification of tracers and NMR strategies, as well as computational tools, are likely to be further developed in this area.

The shift towards human systems and particularly in-cell environments are inevitable since these systems better mimic physiological conditions. It will be important to develop strategies to monitor metabolite-protein interactions in these environments. Studying interaction will contribute to further knowledge of catalytic and allosteric events essential in metabolic regulation.

A more detailed overview of metabolism and its dynamics at the organelle-level - in its cellular compartments - and at the organism level - in specific organs - will be essential to dissect. The interplay between these metabolite pools is indispensable to understand metabolic regulation in health and disease at a systems biochemistry level. While NMR will never make up for its lack of sensitivity, it will enable the study of the many aspects of the spectroscopy of life.
